# Scrub Typhus Leading to Acute Encephalitis Syndrome, Assam, India

**DOI:** 10.3201/eid2301.161038

**Published:** 2017-01

**Authors:** Siraj A. Khan, Trishna Bora, Basanta Laskar, Abdul M. Khan, Prafulla Dutta

**Affiliations:** Regional Medical Research Centre, NE Region, Indian Council of Medical Research, Dibrugarh, Assam, India (S.A. Khan, T. Bora, A.M. Khan, P. Dutta);; Assam Medical College and Hospital, Dibrugarh, Assam, India (B. Laskar)

**Keywords:** Scrub typhus, AES, Assam, India, vector-borne infections, bacteria, acute encephalitis syndrome, meningitis/encephalitis

## Abstract

To determine the contribution of *Orientia tsutsugamushi,* the agent of scrub typhus, as a cause of acute encephalitis syndrome (AES) in Assam, India, we conducted a retrospective study of hospital patients with symptoms of AES during 2013–2015. Our findings suggest that *O. tsutsugamushi* infection leads to AES and the resulting illness and death.

Scrub typhus is a miteborne bacterial disease caused by *Orientia* *tsutsugamushi*. Clinical features generally include fever, headache, and myalgia, with or without eschar/rash (*1*). In India context, scrub typhus was first reported in Assam during World War II (1944–1945) across the India–Myanmar border (*2*). The northeastern region of India then experienced decades without the disease until it reemerged in 2010 (*3*). Assam, a northeastern state in India, is recognized as an endemic zone for acute encephalitis syndrome (AES), especially that caused by Japanese encephalitis virus (JEV). However, the etiology of >50% of the AES cases in Assam remains unrecognized (*4*)*.* In this study, we aimed to determine the contribution of scrub typhus to AES in this region.

We conducted a retrospective study of patients exhibiting symptoms of AES who were admitted during 2013–2015 to Assam Medical College and Hospital, Dibrugarh (a government-funded tertiary care hospital that provides health care for 8 adjoining districts). Serum samples underwent serologic testing with InBios ST Detect IgM ELISA kit (InBios International, Seattle, WA, USA). An optical density of >0.5 was considered positive (*5*). We extracted DNA from the blood samples by using the QIAamp DNA blood mini kit (QIAGEN, Hilden, Germany) and performed nested PCR for amplification of a 56-kDa gene of *Orientia,* targeting a 483-bp fragment (*6*). We compared the sequences obtained with reference strains of *Orientia*.

We enrolled 511 AES case-patients after disease onset (mean 6.23 days; range 1–14 days); 104 (20.3%) had IgM against *O. tsutsugamushi* (suggestive of recent infection) (*7*). Of these 104 patients, 58 (55.7%) were male and 46 (44.2%) were female. Ages ranged from 3 to 80 years (median 25 years). The main clinical features of the 104 IgM-positive case-patients were fever (100%), altered sensorium (100%), headache (67.3%), unconsciousness (55.7%), nausea (40.3%), and neck rigidity (0.9%). None had any record of eschar. Leptospirosis, dengue, and malaria were ruled out for all 104 case-patients. Only 13 (12.5%) of the IgM-positive case-patients were positive for JEV IgM. *Orientia* DNA was detected in 9 (8.6%) of the samples that had IgM against *O. tsutsugamushi*. Percentage similarity of the nucleotide sequences (GenBank accession nos. KU163359, KU163361, KU163362, KU163366, KU163369, KU163370, KU163372, KU163373, and KP067915) demonstrated resemblances with the Karp strain of *Orientia* ranging from 91.9% to 93.7% (Figure). We were able to follow-up 53 of the 104 IgM-positive case-patients and discovered that 26 (49%) died after discharge. These included 4 of the 9 PCR positive case-patients.

**Figure:**
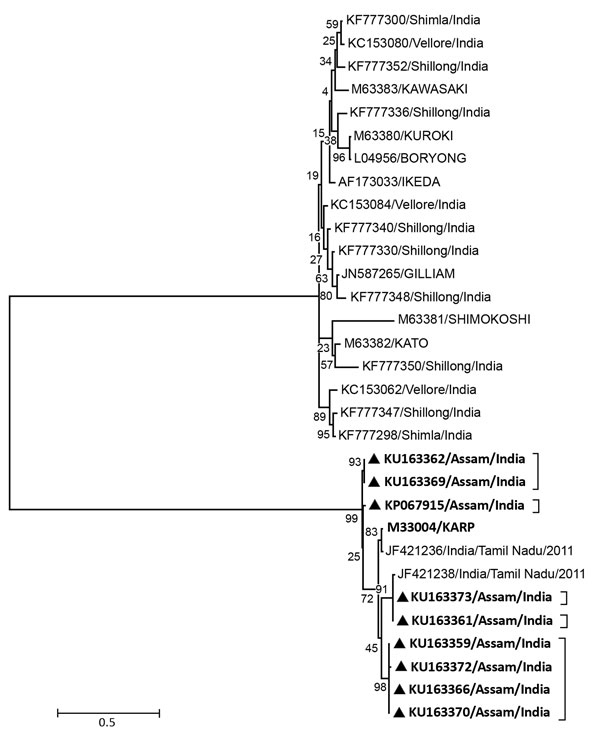
Dendrogram representing *Orientia tsutsugamushi* sequences (black triangles) from patients with acute encephalitis syndrome, Assam, India, 2013–2015. The phylogenetic tree was constructed on a 56-kDa outer membrane protein gene of *O. tsutsugamushi*. The evolutionary history was inferred by using the maximum-likelihood method based on the Tamura 3-parameter model. Our sequences are found within the brackets. Bold indicates the KARP genotype. Scale bars indicate nucleotide substitutions per site.

The high prevalence of illness and death resulting from scrub typhus in this study can be attributed to various reasons. First, because JEV is the predominant etiologic agent of AES in the northeastern region, especially in Assam, health providers generally do not suspect other etiologic factors, including scrub typhus. Moreover, the nonspecific clinical features of scrub typhus in these patients (i.e., absence of eschar/rash) led to diagnostic dilemmas. In addition, JEV infection, West Nile virus infection, and leptospirosis have been identified as key contributors to AES in the northeastern region (*8**,**9*). Treatable bacterial diseases such as leptospirosis and scrub typhus are grossly underestimated because of the low index of suspicion and limited diagnostic facilities, especially in developing countries such as India. Proportion of deaths due to untreated scrub typhus varies across different regions from 0% to 70% (*10*). Our study found a high case-fatality rate of 49%. The absence of distinguishable clinical features among AES-identifying etiologic factors makes differential diagnosis difficult, and thus the condition remains untreated. That clinicians are unaware of the presence of the disease remains a major hindrance to its recognition and successful treatment.

In our study, the highest numbers of scrub typhus cases were recorded during July–September, the peak season for JEV transmission. The northeastern region has a subtropical climatic pattern, and the season (May–August) is also an appropriate period for sowing and harvesting crops. Agricultural lands cover 22% of the northeastern region, with ≈70% of the total population dependent on agriculture for their livelihood. Because our study was retrospective, the occupations of the patients with positive cases were unknown. However, the areas where the followed-up case-patients lived were primarily agrarian.

Our findings suggest that the bacterium that causes scrub typhus, *O. tsutsugamushi*, is a notable etiologic agent that contributes to the occurrence of AES and resulting illness and death. We believe that it is urgent that this neglected, treatable disease be considered when diagnosing those with AES, especially during July–September in AES-endemic regions. Recording of occupation details and ecologic background of the attending patients, especially from those in rural areas, could be a vital source for identifying those with suspected scrub typhus. Increasing awareness among clinicians could lead to prompt diagnosis and effective treatment of this almost-forgotten potential source of AES.
